# Identification of drug targets and potential molecular mechanisms for Wantong Jingu Tablet extract in treatment of rheumatoid arthritis: bioinformatics analysis of fibroblast-like synoviocytes

**DOI:** 10.1186/s13020-020-00339-5

**Published:** 2020-06-05

**Authors:** Zhaodong Li, Fangyuan Qi, Fan Li

**Affiliations:** 1 Department of Pathogen Biology, The Key Laboratory of Zoonosis, Chinese Ministry of Education, College of Basic Medicine, Jilin University, No. 126 Xinmin Street, Changchun, 130021 Jilin China; 2 The Key Laboratory for Bionics Engineering, Ministry of Education, China, Jilin University, Changchun, 130021 Jilin China; 3 Engineering Research Center for Medical Biomaterials of Jilin Province, Jilin University, Changchun, 130021 Jilin China; 4 Key Laboratory for Biomedical Materials of Jilin Province, Jilin University, Changchun, 130021 Jilin China; 5 State Key Laboratory of Pathogenesis, Prevention and Treatment of High Incidence Diseases in Central Asia, Urumqi, Xinjiang China

**Keywords:** Rheumatoid arthritis, Bioinformatics, Fibroblast-like synoviocytes, Wantong Jingu Tablet

## Abstract

**Background:**

Rheumatoid arthritis-fibroblast-like synoviocytes (RA-FLSs) play important roles in pathogenesis of rheumatoid arthritis (RA). Wantong Jingu Tablet (WJT), a mixture of traditional Chinese medicine, is a potentially effective therapy for RA, but its underlying mechanism is unclear. In this study, we explore the effects of WJT on human RA-FLSs and the underlying molecular mechanism.

**Methods:**

The major components of WJT were determined using ultra-high-performance liquid chromatography coupled with quadrupole time-of-flight mass spectrometry (UHPLC-QTOF/MS). Cell proliferative ability was evaluated by CCK-8, colony formation assay, and EdU incorporation assay. Cell apoptotic capacity was examined by caspase-3 and caspase-9 activity test. Protein levels of Bax and Bcl-2 were investigated by western blotting. High-throughput sequencing and bioinformatics analysis were conducted to screen and identify targeted genes, followed by identification by qRT-PCR and western blotting.

**Results:**

In this study, we have identified 346 compounds in WJT. Our results showed that WJT inhibited the RA-FLSs proliferation, and promoted apoptosis in a dose- and time-dependent manner. More importantly, 184 differentially expressed genes (DEGs) has been screened after WJT treatment based on DEGSeq2 and 278 DEGs was identified by DEGSeq2 combined with WGCNA. Then, 10 hub genes were identified based on two different analyses, while the expression levels of only *SMC3*, *THOC1*, *BUB1*, and *STAG2* were decreased after WJT treatment, which was identical to the sequencing profiles.

**Conclusions:**

WJT exerted its anti-proliferation and pro-apoptosis effects possibly through suppressing the expression of *SMC3*, *THOC1*, *BUB1*, and *STAG2* in RA-FLSs. Thus, therapeutics targeting these genes may be a promising strategy for rescuing RA.

## Background

Rheumatoid arthritis (RA) is a systemic autoimmune disease characterized by chronic inflammation, synovial hyperplasia, and bone hyperplasia and destruction [1, 2]. Synoviocytes are the major cells in the synovial membrane, and these are the terminal targets in the pathogenesis of RA [3, 4]. Synovial hyperplasia, which contributes to the progression and development of RA, mainly results from the active proliferation and aggressive tumor-like characteristics of rheumatoid arthritis-fibroblast-like synoviocytes (RA-FLSs) [5, 6]. Therefore, inhibition of the proliferation of RA-FLSs has potential as a treatment for RA.

Wantong Jingu Tablet (WJT), the prescription drug approved by the Chinese government for RA treatment (Approval Number: Z20025183), is an herbal compound containing 25 herbal medicines with many pharmaceutical effects. It exerted curative effects on treating cervical spondylotic myelopathy, a serious degenerative disease, and the total effective rate was up to 83.3% [7]. Recently, a study has found that WJT had clinical curative effect in treatment of periarthritis, significantly relieving shoulder pain and improving shoulder function [8]. Moreover, WJT could protect against collagen-induced arthritis in rats by disturbance of gene expression levels and induction of synoviocytes apoptosis [9]. These studies suggest that WJT has pharmaceutically potential roles in restoring the pathogenesis and progression of RA. Therefore, the effects of WJT on human RA-FLSs and the underlying mechanism will be further investigated in this work.

Recent developments in bioinformatics and molecular biology have led to high-throughput sequencing (RNA-seq), which can simultaneously measure the expression of many genes [10, 11]. Many researchers have used these techniques to characterize the mechanisms of various diseases and to identify potential therapeutic targets [12, 13]. Therefore, we will apply the RNA-seq technology and corresponding bioinformatics analysis to identify the molecular mechanisms, biological processes, and potential drug targets in this study. Given the high false-positive rate associated with analysis by DESeq2 or edgeR, we use a combination analysis of DESeq2 and weighted gene co-expression network analysis (WGCNA) to screen for hub genes.

## Materials and methods

### Preparation of samples and solutions

Samples of WJT powder were provided by Jilin Wantong Pharmacy Group Company (Tonghua, China). The traditional Chinese medicines containing in WJT were shown in Additional file 1: Table S1. An alcohol-water dual extraction method was applied to ensure complete extraction. First, a 2.5 kg sample was dissolved in 25 L of 75% ethanol for 7 days, and the sample was then extracted via reflux to obtain filtrates. This above procedure was repeated, and the filtrates were then combined. The collected filtrates were concentrated under vacuum conditions until there was no scent of alcohol. The final extracts were packed separately, sealed, and stored at − 80 °C. Extracts were dissolved with 0.1% DMSO at different concentrations prior to experiments.

For pharmaceutical analysis, a sample extract (1 g) was placed in a 25 mL methanol solution. Then, the solution was passed through a 0.22 µm filter prior to ultra-high-performance liquid chromatography coupled with quadrupole time-of-flight mass spectrometer (UHPLC-QTOF-MS) analysis.

### UHPLC-QTOF-MS

UHPLC-QTOF-MS was conducted on an Agilent 1290 ultra-high definition accurate mass QTOF spectrometer with UHPLC (Agilent Technologies, AB Sciex, CA, USA). A UPLC C18-column (2.1 mm × 100 mm, ID 1.7 μm, ACQUITY UPLC^®^ BEH; Waters, Milford, MA, USA) was used for separation, together with a C18-pre-column (2.1 mm × 5 mm, ID 1.7 μm, VanGuardTM BEH; Waters) at room temperature (20 °C). The mobile phase consisted of 0.1% formic-acid–water (A) and 0.1% formic-acid–acetonitrile (B), with the following optimized linear gradient elution: 0–3.5 min, 5% B; 3.5–6 min, 1% B; 6–12 min, 30% B; 12–12.5 min, 70% B; 12.5–22 min, 100% B. The injection volume was 5 μL and the flow rate was of 400 μL/min. Mass spectra were acquired in positive mode and negative mode. Data analysis was performed using Progenesis QI. Retention time correction, peak identification, peak extraction, peak integration, and peak alignment were optimized by the software. The corresponding Chinese medicine metabolism library was also queried to identify compounds. Then, matching between the self-built secondary mass spectrometry database and the corresponding fragmentation regularity as used to identify the peaks based on MS/MS data.

### Cell culture

Human RA‐FLSs were obtained from BeNa Culture Collection (BeiJin, China) and were cultured in high glucose Dulbecco’s Modified Eagle’s medium (DMEM; Hyclone, Logan, Utah, USA) that was supplemented with 10% (v/v) fetal bovine serum (FBS; Gibco, Grand Island, NY, USA), 100 U/mL penicillin (MRC, Jintan, China), and 100 mg/mL streptomycin (MRC) at 37 °C and 5% CO_2_. RA-FLSs obtained from passages three to six were used for experiments.

### Cell viability and proliferation assays

RA-FLSs were seeded in 96-well plates 4 × 10^3^/cells. After overnight, RA-FLSs were treated with various concentrations (0, 1, 2, 3, or 4 mg/mL) of WJT extract for different time (0, 12, 24, 36, 48, and 72 h). Then, cell viability was measured by the Cell Counting Kit (CCK-8; Beyotime, Shanghai, USA) according to manufacturer’s instructions. Controls were treated with 0 mg/mL WJT extract. In addition, RA-FLSs were plated in 6-well plates 0.5 × 10^3^/cells. After incubation with WJT extract for 24 h and 48 h, cells were cultured for another 2 weeks. Then, cells were stained with Giemsa’s dye solution and the number of colonies was counted using Image J software (Version 1.8.0). For the 5-ethynyl-2-deoxyuridine (EdU) incorporation assay (Solarbio, Beijing, China), cell proliferation was assessed following the manufacturer’s protocol after treatment with WJT extract.

### Cell morphology

The cell morphology was examined using an optical microscope. In addition, cells were collected by centrifugation (1000 rpm for 5 min) and then fixed in a 2.5% glutaraldehyde solution at 4 °C for 10 h. The fixed cells were rinsed three times with PBS, and then dehydrated in a graded series of ethanol solutions (30%, 50%, 70%, 90% and 100%) at 1 min intervals. After 12 h of freeze-drying, the cells were covered by cathodic spraying, for observation by scanning electron microscopy (SEM).

### Cell apoptosis assay

Enzyme linked immunosorbent assay (ELISA) kits (Abcom, Cambridge, UK) were utilized to measure the activity of caspase-3 and caspase-9 following the manufacturer’s guidelines. Protein levels of Bax and Bcl-2 were evaluated by western blotting. And primary antibodies against Bax and Bcl-2 were from Abcom.

### Profiling of mRNA expression

After incubation with 3 mg/mL WJT extract for 24 h and 48 h, RNA in RA-FLSs was extracted for sequencing. The NEBNext^®^ UltraTM RNA Library Prep Kit for Illumina^®^ (NEB, USA) was used to profile the expression of mRNAs. Quality control and quantification of gene expression were also performed. The DESeq2 R package (1.16.1) was used to perform the differential expression analysis of the different treatment groups (3 replicates per group). The Benjamini–Hochberg approach was used to adjust *P*-values and decrease the false discovery rate. Subsequently, the significant DEGs (adjusted *P* < 0.05 and |log_2_(fold-change)| > 1) were identified by DESeq2. The STEM software (version 1.3.12) was utilized to conduct the Series-Cluster analysis of expression profiles of DEGs. A co-expression network was created by WGCNA using the R package (Version 1.68). Then, Gene Ontology (GO) and Kyoto Encyclopedia of Genes and Genomes (KEGG) enrichment analysis of DEGs were determined using the clusterProfiler R package. The protein–protein interaction (PPI) networks were constructed using a search tool for the retrieval of interacting genes (STRING) (http://www.string-db.org/). These results were visualized using Cytoscape software (version 3.6.0).

### Quantitative real-time polymerase chain reaction (qRT-PCR)

Total RNA was extracted from RA-FLSs using a Total RNA Extraction Kit (Solarbo, Beijing, China), and reverse transcription was performed using a first-strand cDNA synthesis kit (Invitrogen, Carlsbad, CA, USA), following the manufacturer’s guidelines. Premix Ex Taq SYBR Green PCR (TaKaRa, Dalian, China) was used to conduct real-time PCR on an ABI PRISM 7300 (Applied Biosystems, Foster City, CA, USA) following the manufacturer’s protocols. Table 1 shows the sequences of the primers, and GAPDH was used as the internal control.

**Table 1:** Primer sequences used for qRT-PCR

Gene	Primer	Sequence (5′→3′)
*BUB1*	Forward	AGCCCAGACAGTAACAGACTC
	Reverse	GTTGGCAACCTTATGTGTTTCAC
*THOC1*	Forward	GAAAAATGAAGGTTGCCCAAGTT
	Reverse	TTGTCTCTGATTTACAGGCTTCC
*SMC3*	Forward	AACATAATGTGATTGTGGGCAGA
	Reverse	TCCTTTTTGGCACCAATAACTCT
*STAG2*	Forward	TCCTTCTGGTCCAAACCGAAT
	Reverse	ACCGACTGCATAGCACTCTTG
*ORC4*	Forward	AGATTTTCTCACCGGCAGATACA
	Reverse	AGCAAGCATCAATAGCATGTGT
*TTK*	Forward	TCATGCCCATTTGGAAGAGTC
	Reverse	CCACTTGGTTTAGATCCAGGC
*ATR*	Forward	TCCCTTGAATACAGTGGCCTA
	Reverse	TCCTTGAAAGTACGGCAGTTC
*BRCA1*	Forward	GAAACCGTGCCAAAAGACTTC
	Reverse	CCAAGGTTAGAGAGTTGGACAC
*EIF3A*	Forward	ACTCAGGATCGTACTGACAGATT
	Reverse	ATGGTACAGGCGCTCTACTCT
*PNN*	Forward	CTGGTCCTGGTGGAGGTAGA
	Reverse	TGTGCGCTCTTTGGAGGTAG
*GAPDH*	Forward	GGAGCGAGATCCCTCCAAAAT
	Reverse	GGCTGTTGTCATACTTCTCATGG

### Western blot analysis

After extraction of total protein from RA-FLSs, the protein concentration was determined using the Bradford method (Beyotime). Equal amounts of protein lysates (20 μg per lane) were separated by 8–12% SDS-PAGE gels, and the proteins were then transferred to 0.45 µm polyvinylidene fluoride (PVDF) membranes (ThermoFisher, Waltham, MA, USA). The membranes were blocked using 5% BSA (Solarbio) and incubated with primary antibodies (all from Abcom) at 4 °C overnight. Then, the PVDF membranes were treated with horseradish peroxidase-conjugated secondary antibody (Bioss) at room temperature for 2 h. Protein bands were visualized using a Tanon 5200 (Tanon, Shanghai, China).

### Statistical analysis

Data are presented as means ± standard deviations (SDs). Student’s t-test was used to compare groups, and all statistical analyses were conducted using SPSS version 20.0. All experiments were performed at least three independent times. The significance of statistical differences are expressed as *(*P* < 0.05), **(*P* < 0.01), and ***(*P* < 0.001).

## Results

### Major components of WJT identified by UHPLC-QTOF-M*S*

**Fig. 1:**
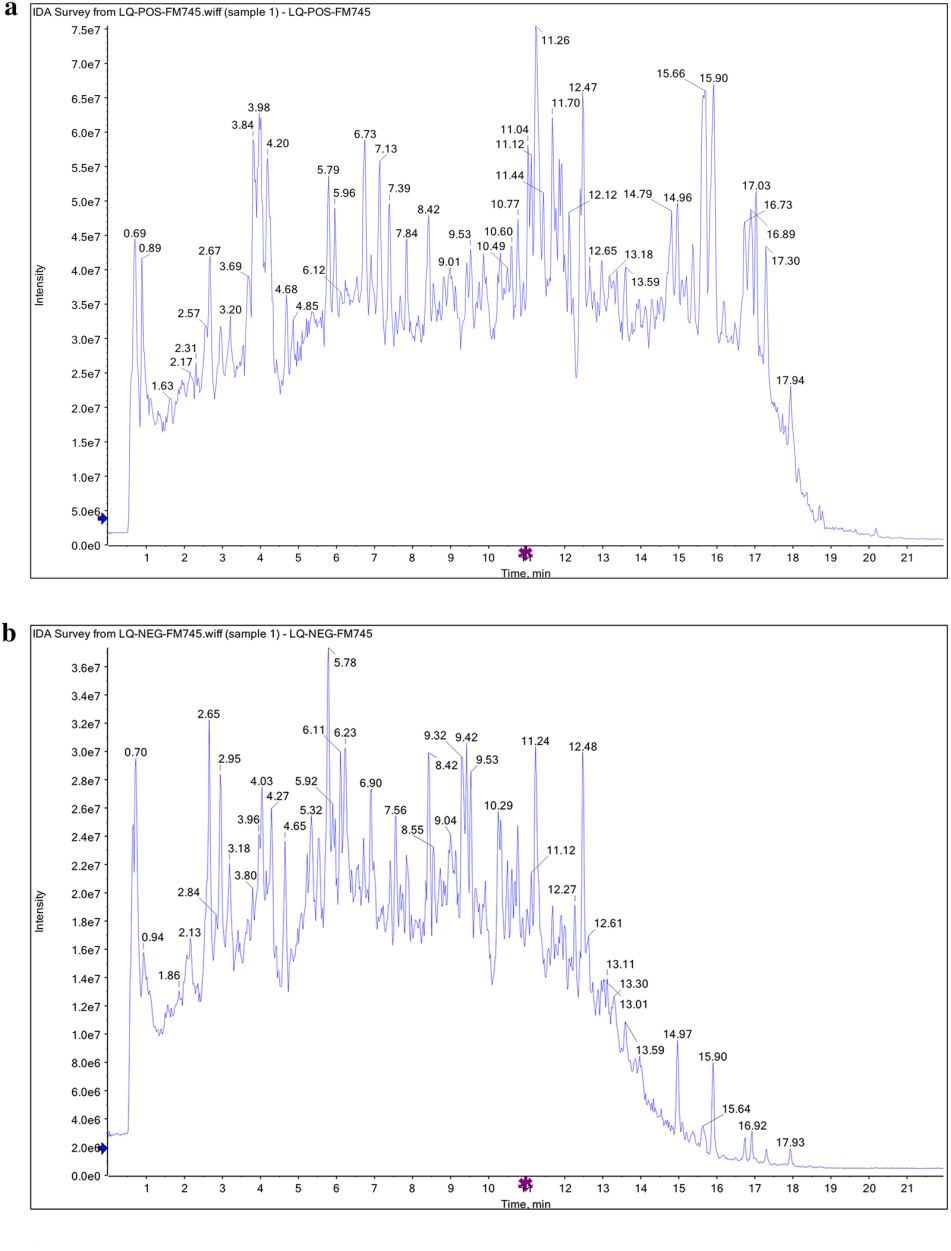
Total ion chromatography (TIC) of WJT extract. Data were captured by UHPLC-QTOF-MS in positive mode and indicated 169 peaks (**a**). Data were captured in negative mode and indicated 177 peaks (**b**). n = 3 per group

We first determined the components of WJT extract using UHPLC-QTOF-MS. The results indicated 169 peaks in positive mode (Fig. 1, Additional file 1: Table S2) and 177 peaks in negative mode (Additional file 1: Table S3).

### WJT extract inhibited the viability and proliferation of RA-FLSs

**Fig. 2:**
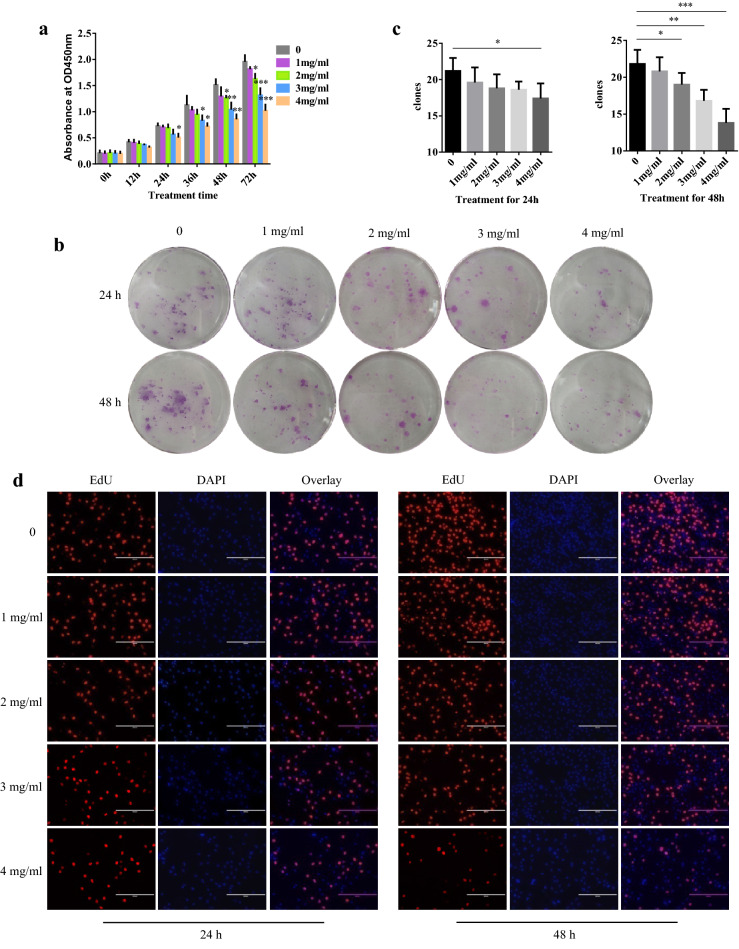
Effect of WJT extract on proliferation of RA-FLSs. RA-FLSs were treated with different concentrations of WJT extract for 0–72 h, and the cell viability was assayed using CCK8 assay (**a**). Cell proliferation was determined by the colony formation (**b**, **c**) and the EdU analysis (×100 magnification) (**d**, **e**). Values were expressed as mean ± SD (n = 3). *P < 0.05, **P < 0.01, ***P < 0.001 vs. control. Control was treated with 0 mg/mL WJT extract

RA-FLSs were exposed to 0–4 mg/mL WJT extract for 0, 12, 24, 36, 48, and 72 h, and then CCK-8 assays were performed. The results indicated that WJT inhibited the RA-FLSs viability in a dose- and time-dependent manner (Fig. 2a). In addition, the suppressive effects of WJT on RA-FLAs were verified by colony formation (Fig. 2b, c) and EdU analysis (Fig. 2d).

### WJT induced apoptosis of RA-FLSs

**Fig. 3:**
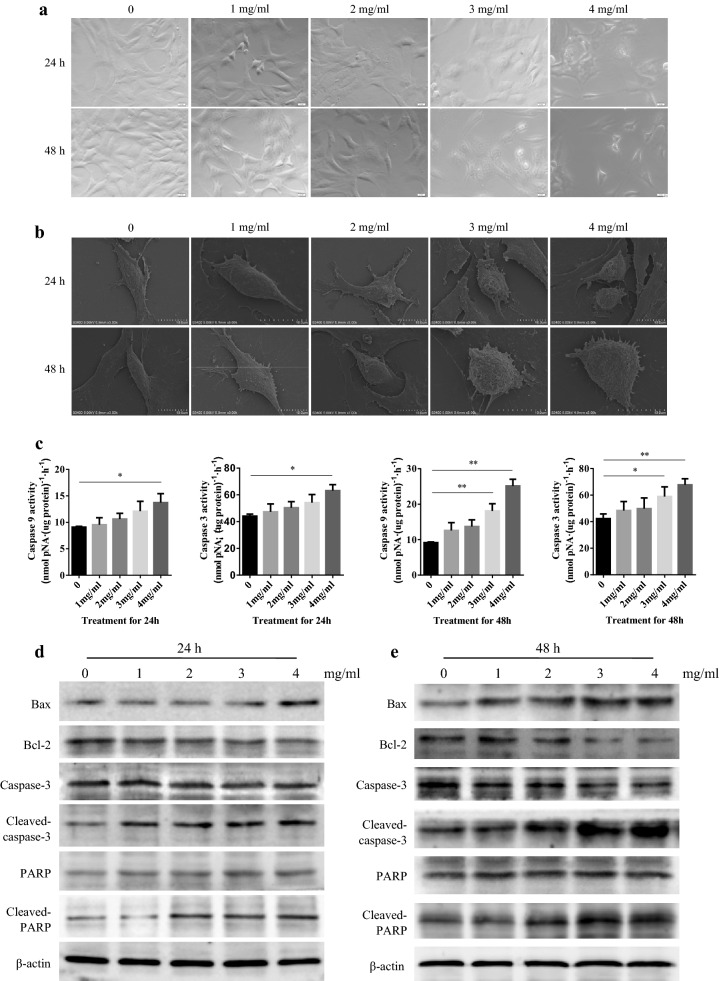
Effect of WJT extract on apoptosis of RA-FLSs. RA-FLSs were treated with 0–4 mg/mL WJT extract WJT extract for 24 h and 48 h. Representative images were illustrated by the optical microscope (**a**, ×200 magnification) and scanning electron microscope (**b**, ×3000 magnification). ELISA was performed to measure the *caspase*-*3* and *caspase*-*9* activities (**c**). Western blotting was performed to measure the protein levels of Bax, Bcl-2, caspase-3, cleaved-caspase-3, PARP and cleaved-PARP (**d**, **e**). Values were expressed as mean ± SD (n = 3). *P < 0.05, **P < 0.01 vs. control

WJT supplement markedly altered the RA-FLSs morphologies in a dose- and time-dependent manner (Fig. 3a, b). Additionally, the caspase-9 and caspase-3 activities were promoted following treatment with WJT extract for 24 h and 48 h (Fig. 3c), thereby indicating that WJT supplement induced RA-FLSs apoptosis. And the pro-apoptotic effects of WJT extract on RA-FLSs were illustrated by western blotting (Fig. 3d, e).

### Identification of hub genes by DESeq2

**Fig. 4:**
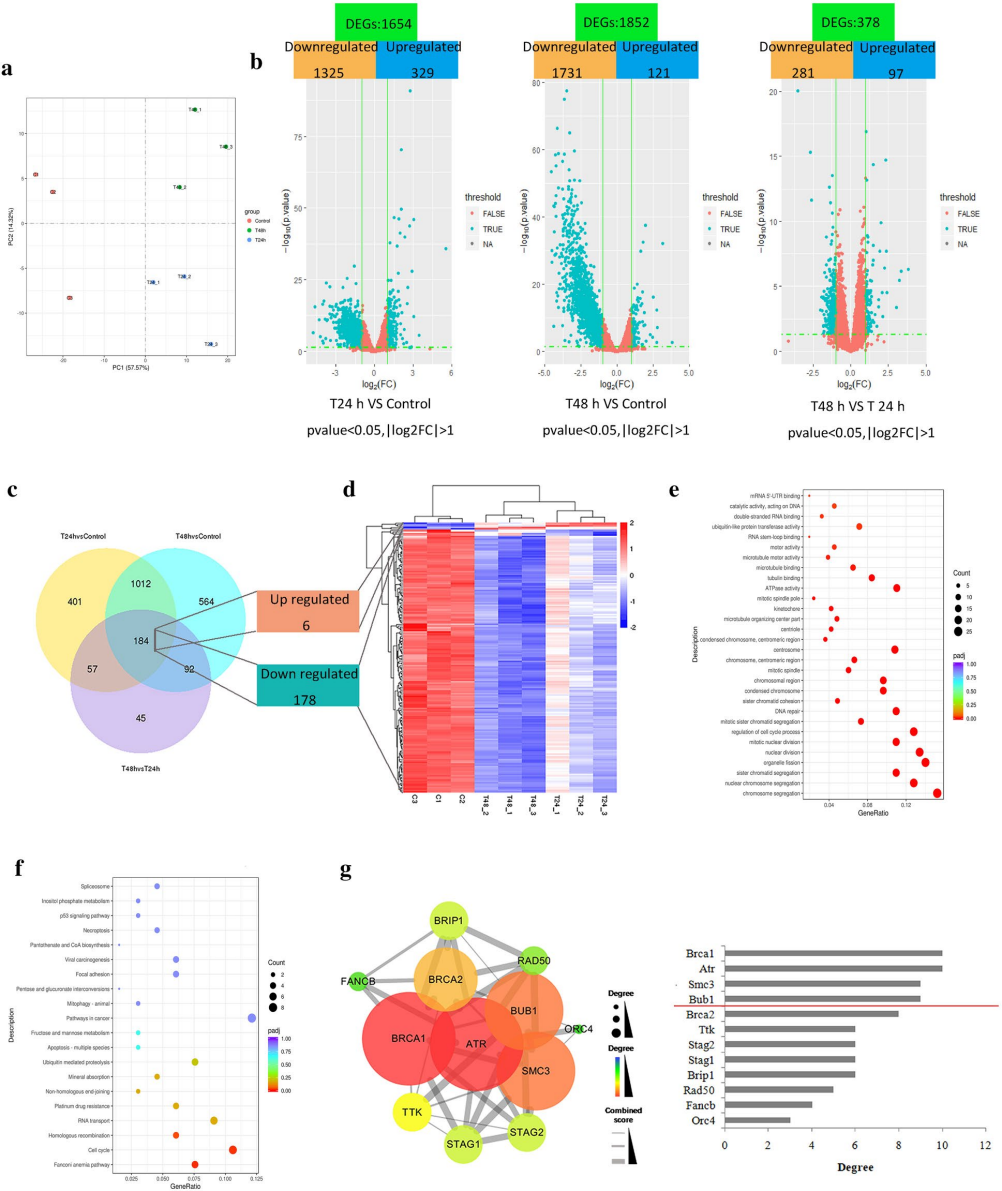
Identification of hub genes through DESeq2. RA-FLSs were exposed to 3 mg/mL of WJT extract for 24 h and 48 h, and RNA was extracted for RNA-seq. Principal component analysis (PCA) of the mRNA expression among different groups (**a**). Volcano plot of the differentially expressed genes (DEGs) among different groups (**b**). Red DEGs: |log_2_(fold-change)| < 1; turquoise DEGs: |log_2_(fold-change)| > 1. Venn diagram of the DEGs among different groups, showing 184 common DEGs (6 upregulated and 178 downregulated) (**c**). Heat map of the 184 common DEGs among samples (**d**). Dotplot of the GO enrichment analysis (**e)** and the KEGG enrichment analysis (**f**) for the 184 DEGs. Protein-protein interaction (PPI) network of the DEGs in the significantly enriched pathways, and the node degrees in PPI relationship (**d**). n = 3 per group

The PCA results revealed that the main characteristics of the three groups can be easily distinguished, indicating the mRNA expression among groups were significantly different (Fig. 4a). The volcano plots were used to illustrate differentially expressed genes (DEGs) among different groups (Fig. 4b), and there were 184 common DEGs among the three compared groups, including 6 upregulated genes and 178 downregulated genes (Fig. 4c, d).

Figure 4e indicated the 184 DEGs were significantly enriched in chromosome segregation, nuclear chromosome segregation, sister chromatid segregation, and so on. The 12 DEGs were significantly enriched in fanconi anemia pathway, cell cycle, and homologous recombination (Fig. 4f). Additional file 1: Tables S4 and S5 provided more details of the GO and KEGG enrichment analysis. Then, the 12 DEGs were used to construct a protein–protein interaction (PPI) network by STRING database and Cytoscape software (Fig. 4g). Among these genes, *BRCA1*, *ATR*, *SMC3* and *BUB1* possessed the highest node degree, which was identified as hub genes.

### Identification of hub genes by the combined use of DESeq2 and WGCNA

**Fig. 5:**
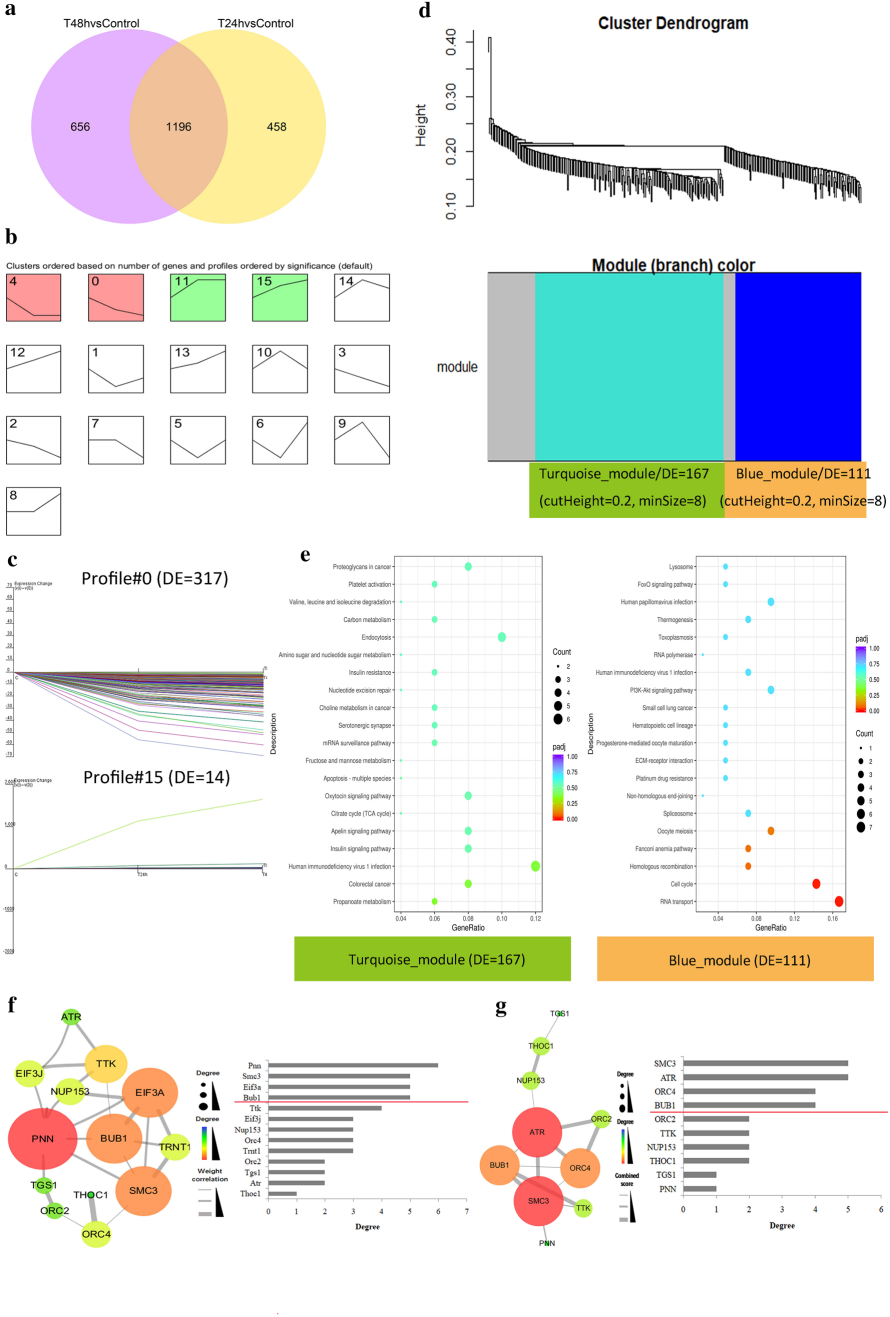
Identification of hub genes by the combined use of DESeq2 with WGCNA. Venn diagram of the DEGs among different groups, showing 1196 common DEGs (**a**). Time series cluster analysis of 1196 DEGs, confirming 16 model profiles (**b**). 331 DEGs from the two model profiles (profile#0 and profile#15), bearing a positive or negative correlationship with the treatment time (**c**). Clustering of the 331 DEGs by WGCNA, and identification of two co-expression modules: turquoise module and blue module (**d**). Dotplot of the KEGG pathway analysis for the turquoise and blue modules (**e**). Co-expression network of DEGs in the significantly enriched pathways was constructed by Cytoscape software and the node degrees were analyzed (**f**). PPI network of DEGs in the significantly enriched pathways, and the node degrees in PPI relationship (g). n = 3 per group

There were 1196 common DEGs among the two compared groups (Fig. 5a). Time series cluster analysis suggested 4 gene expression profile patterns (NO. 0, 4, 11 and 15) were significantly changed in gene expression (Fig. 5b). And the NO. 0 and 15 patterns presented positive or negative correlations with treatment time, 317 genes and 14 genes, respectively (Fig. 5c). The 331 DEGs were clustered by WGCNA and 2 distinct co-expression modules, a “Turquoise module” (167 DEGs) and a “Blue module” (111 DEGs) were already identified (Fig. 5d).The clustered DEGs were significantly enriched in RNA transport and cell cycle (Fig. 5e, Additional file 1: Tables S6 and S7). Then, we constructed a weighted gene co-expression network for these significantly enriched DEGs, and genes with the top 4 degrees in the network were defined as “hub genes”: *PNN*, *SMC3*, *EIF3A*, and *BUB1* (Fig. 5f**)**. A PPI network of these genes indicated the genes with the first four degrees in the PPI network (hub genes) were: *SMC3*, *ATR*, *ORC4*, and *BUB1* (Fig. 5g).

### Hub genes identified by qRT-PCR and western blotting

**Fig. 6:**
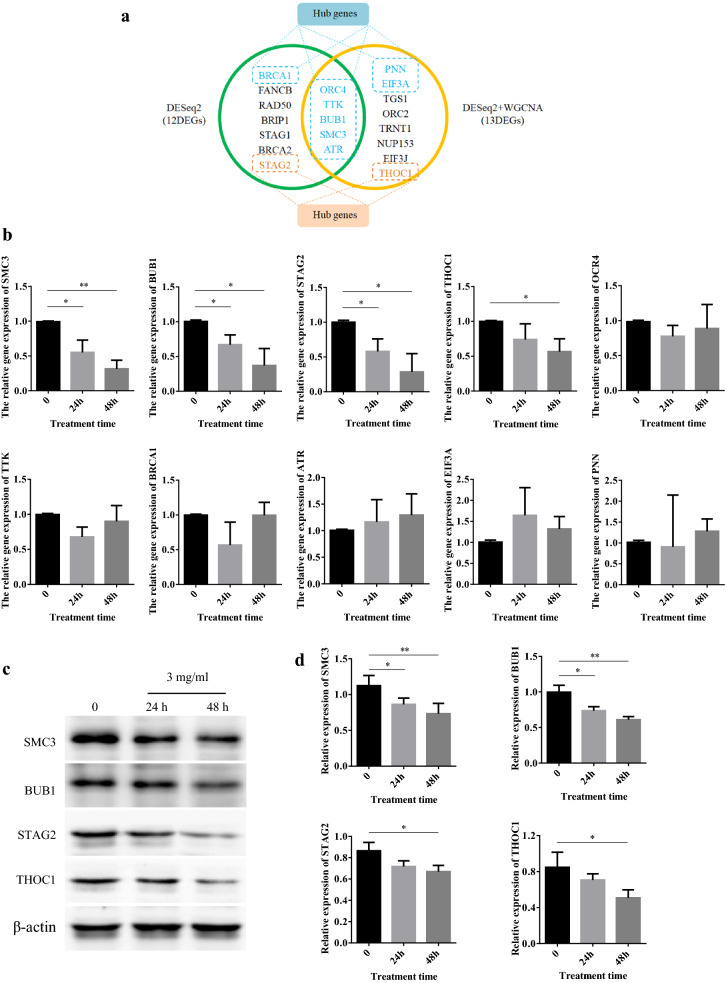
Validation of hub genes by qRT-PCR and western blotting. Venn diagram of the major DEGs from two different analysis methods (**a**). qRT-PCR analysis for the hub genes (**b**). Western blotting was performed to measure the protein levels of the hub genes (**c**, **d**). Values were expressed as mean ± SD (n = 3). *P < 0.05, **P < 0.01 *vs.* control

There were five overlapping genes, *BUB1*, *ORC4*, *SMC3*, *TTK*, and *ATR*, from significantly enriched KEGG pathways identified by two different analysis methods, which was defined as hub genes (Fig. 6a). In addition, *THOC1* and *STAG2* played important roles in cell proliferation and apoptosis. Thus, we also classified *THOC1* and *STAG2* as hub genes in the present study. qRT-PCR, used for hub genes validation, indicated that *SMC3*, *BUB1* and *STAG2* were significantly down-regulated after 3 mg/mL WJT treatment, consistent with the RNA-seq results (Fig. 6b). *THOC1* expression had a tendency for reduction at 24 h following WJT treatment, and its expression was significantly suppressed after 48 h. The protein levels of SMC3, BUB1, STAG2, and THOC1 were further examined by western blotting, which suggested that WJT markedly reduced the expression of SMC3 and BUB1 in RA-FLSs after 3 mg/mL WJT treatment, and the expression of STAG2 and THOC1 were also significantly suppressed after 48 h (Fig. 6c, d).

## Discussion

Rheumatoid arthritis (RA) is an autoimmune disease characterized by inflammation of the joints, that ultimately progresses to deformities and disability [14, 15]. An increasing number of RA patients and those with other diseases are selecting natural products to satisfy their healthcare needs [16, 17]. WJT is a type of traditional Chinese medicine (TCM) that is a mixture of herbal compounds, and has been used for clinical treatments for many years. However, little is known about the ingredients of WJT. Therefore, UHPLC-QTOF-MS analysis was performed to identify the major components in WJT. The magnoflorine, a component of WJT, can ameliorate the strong pulmonary inflammation via suppressing NF-κB and MAPK activation [18], and it also induces the cancer cells apoptosis and autophagy through AKT/mTOR and p38 signaling pathways [19]. Meanwhile, the loganin is an iridoid glycoside in WJT and has been reported to be responsible for inhibiting inflammation by regulating JAK/STAT3 signaling pathway and TLR4/TRAF6/NF-κB axis [20, 21]. Thus, the UHPLC-QTOF-MS results might contribute to study and understand the mechanisms for WJT in rescuing RA, and further investigate the active ingredients involved in the treatment.

Synoviocytes are the major cells in synovial tissues, and pathological alterations of these cells occur during the onset and pathogenesis of RA [22, 23]. Abnormal proliferation of RA-FLSs can promotes immune cells to secrete numerous pro-inflammatory cytokines and proteins that function in innate immunity and matrix-degradation, which further promote the pathogenesis of RA [24, 25]. Thus, inhibition of RA-FLS proliferation is a promising approach for treatment of for RA. Alteration of RA-FLSs apoptosis is another potential target for treatment of RA [26, 27]. It’s well known that pro-apoptotic proteins, such as Bak and Bax, activate apoptosis, while anti-apoptotic Bcl-2 family proteins, such as BclXL and Bcl-2, inhibit apoptosis [28, 29]. A increased ration of Bax/Bcl-2 could result in the release of cytochrome c from mitochondria and activation of caspase-3 and caspase-9, thereby inducing apoptosis through the mitochondria-associated apoptotic pathway [30]. In this study, we observed that Bax/Bcl-2, caspase-3 activity and caspase-9 activity were upregulated after WJT treatment.

In current study, we initially identified 10 hub genes from tow different bioinformatics analysis, including *ORC4*, *SMC3*, *BUB1*, *STAG2*, *RAD50*, *TPR*, *TRNT1*, *ATM*, *BRCA1*, and *THOC1.* Our further analysis of these hub genes by qRT-PCR and western blotting indicated that only the expression levels o*f SMC3*, *BUB1*, *STAG2*, and *THOC1* were consistent with the RNA-seq results. The four genes were mainly enriched in two pathways such as Cell cycle (*SMC3*, *BUB1* and *STAG2*) and RNA transport (*THOC1*).

Budding uninhibited by benzimidazoles 1 (BUB1), a mitotic checkpoint serine/threonine kinase, is responsible for chromosome segregation, recruitment of the mitotic checkpoint complex, and activation of the spindle checkpoint [31, 32]. Recent research indicated that BUB1 functioned as an oncogene in different tumors, in that it promoted cancer cell proliferation and invasion [33]. Other studies have also reported correlations between BUB1 expression and tumor proliferation [31, 34].

Stromal Antigen 2 (STAG2) is a subunit of the cohesin complex, a multi-protein ring comprised of two proteins that are responsible for structural maintenance of chromosomes (SMC1 and SMC3) and two non-SMC proteins (RAD21 and STAG1/2) [35]. Among the four subunits, STAG2 predominantly functions in sister chromatid cohesion and segregation. Some recent research demonstrated that a greater abundance of the STAG2 subunit in the cohesin complex assured that sister chromatid cohesion is maintained during the S phase (DNA replication) [36, 37].

SMC3, a subunit of cohesion complex, is responsible for sister chromatid cohesion during mitosis and meiosis in eukaryotes. It has a function similar to that of STAG2. And SMC3 inhibited cell apoptosis and promoted cell growth, migration, and invasion [38, 39]. Another report found that PCI34051, a histone deacetylase 8 (HDAC8)-specific inhibitor, blocked SMC deacetylation and thus blocked cell cycle progression and cell survival in MCF7 breast cancer cells [36].

The human protein THO complex 1 (THOC1) was originally identified as a nuclear matrix component that binds to the tumor suppressor retinoblastoma (RB) protein [40, 41]. Recent research found that high levels of THOC1 were associated with tumor size and aggressiveness in breast and lung cancer cells, and that down-regulation of THOC1 was essential for the NO-mediated cytotoxicity induced by CCL-34 in cancer cells [40].

Taken together, these data suggest that *BUB1*, *STAG2*, *SMC3*, and *THOC1* function in cell growth, proliferation, and tumorigenesis and thus play crucial roles in cancer development. These findings are in agreement with our results. The downregulation of these four hub genes in RA-FLSs following treatment with WJT suggests that overexpression of these genes might contribute to the onset and progression of RA.

## Conclusion

In summary, we found that WJT extract inhibited RA-FLSs proliferation and promoted apoptosis in a dose- and time-dependent manner. In addition, *BUB1*, *STAG2*, *SMC3*, and *THOC1* were identified as hub genes with integrated bioinformatics analysis. The expression levels of these four hub genes in RA-FLSs were identified by qRT-PCR and western blotting to decrease over time with WJT treatment. Therefore, WJT exerted its anti-proliferation and pro-apoptosis effects might through suppressing the expression of *SMC3*, *THOC1*, *BUB1*, and *STAG2* in RA-FLSs. However, the current study is performed based on bioinformatics methods and the conclusions remain to be confirmed by corresponding experiments. While the 346 compounds in WJT were identified, the active components were unknown. Thus, further experimental study is required to verify the present findings and screen the functional components.

## Supplementary information

**Additional file 1: Table S1.** The traditional Chinese medicine containing in WJT. **Table S2.** The compounds in WJT extract identified by UHPLC-QTOF-MS in positive mode. **Table S3.** The compounds in WJT extract identified by UHPLC-QTOF-MS in negative mode. **Table S4.** Five major functions of 184 DEGs in three different categories of GO analysis. **Table S5.** Ten major KEGG pathways among the 184 DEGs. **Table S6.** Ten major KEGG terms based on DEGs in the Turquoise module. **Table S7.** Ten major KEGG terms of DEGs in the Blue module.

## Data Availability

The datasets generated for this study are available on request to the corresponding author.

## Abbreviations

RA-FLS: Rheumatoid arthritis-fibroblast-like synoviocyte
WJT: Wantong Jingu Tablet
UHPLC-QTOF/MS: Ultra-high-performance liquid chromatography coupled with quadrupole time-of-flight mass spectrometry
TIC: Total ion chromatography
DEGs: Differentially expressed genes
RNA-seq: High-throughput RNA sequencing
PCA: Principal component analysis
GO: Gene Ontology
KEGG: Kyoto Encyclopedia of Genes and Genomes
PPI: Protein–protein interaction
DESeq2: Differential gene expression analysis based on the negative binomial distribution
WGCNA: Weighted gene co-expression network analysis
