# Supplementary material for: Identification of drug targets and potential molecular mechanisms for Wantong Jingu Tablet extract in treatment of rheumatoid arthritis: bioinformatics analysis of fibroblast-like synoviocytes
Source: Chin Med. 2020 Jun 5;15:59. doi: 10.1186/s13020-020-00339-5 (PMC7275334; doi:10.1186/s13020-020-00339-5)
Supplement: Supplementary file 1 — Additional file 1: Table S1. The traditional Chinese medicine containing in WJT. Table S2. The compounds in WJT extract identified by UHPLC-QTOF-MS in positive mode. Table S3. The compounds in WJT extract identified by UHPLC-QTOF-MS in negative mode. Table S4. Five major functions of 184 DEGs in three different categories of GO analysis. Table S5. Ten major KEGG pathways among the 184 DEGs. Table S6. Ten major KEGG terms based on DEGs in the Turquoise module. Table S7. Ten major KEGG terms of DEGs in the Blue module. [file 13020_2020_339_MOESM1_ESM.docx]

**Table S1.** The traditional Chinese medicine containing in WJT.

| **NO.** | **Chinese Pinyin name** | **Latin name** |
| --- | --- | --- |
|  | **Herbal medicine** | |
| 1 | Chuanwu | Aconitum carmichaelii Debeaux |
| 2 | Caowu | Aconitum kusnezoffii Rchb. |
| 3 | Maqianzi | Strychnos nux-vomica L. |
| 4 | Yinyanghuo | [Epimedium brevicornu Maxim.](http://www.theplantlist.org/tpl1.1/record/kew-2791222) |
| 5 | Niuxi | Achyranthes bidentata Blume |
| 6 | Qianghuo | Notopterygium incisum K.C.Ting ex H.T.Chang |
| 7 | Guanzhong | Dryopteris crassirhizoma Nakai |
| 8 | Guanhuangbo | Phellodendron amurense Rupr. |
| 9 | Xuduan | [Dipsacus asper Wall. ex C.B. Clarke](http://www.theplantlist.org/tpl1.1/record/tro-11200008) |
| 10 | Wumei | Prunus mume (Siebold) Siebold & Zucc. |
| 11 | Xixin | Asarum sieboldii Miq. |
| 12 | Mahuang | Ephedra sinica Stapf |
| 13 | Guizhi | Cinnamomum cassia (L.) J.Presl |
| 14 | Honghua | Carthamus tinctorius L. |
| 15 | Ciwujia | Acanthopanax senticosus (Rupr. & Maxim.) Harms |
| 16 | Jinyinhua | Lonicera japonica Thunb. |
| 17 | Sangjisheng | Taxillus sutchuenensis (Lecomte) Danser |
| 18 | Gancao | Glycyrrhiza uralensis Fisch. |
| 19 | Gusuibu | Davallia mariesii T. Moore ex Baker |
| 20 | Difengpi | Illicium difengpi B.N.Chang |
| 21 | Moyao | Commiphora myrrha (Nees) Engl. |
| 22 | Hongshen | Panax ginseng C.A.Mey. |
|  | **Insect medicine** | |
| 1 | Wushaoshe | Zaocys dhumnades |
| 2 | Lurong | cornu cervi pantotrichum |
| 3 | Dilong | Geosaurus |

**Table S2.** The compounds in WJT extract identified by UHPLC-QTOF-MS in positive mode.

| **NO.** | **Name** | **RT (min)** | **Mass (m/z)** | **Formula** |
| --- | --- | --- | --- | --- |
| 1 | Magnoflorine | 342.1692 | 3.9871 | C20H24NO4 |
| 2 | Obacunone | 455.2051 | 10.6030 | C26H30O7 |
| 3 | Limonin | 471.2004 | 9.5320 | C26H30O8 |
| 4 | Loganic acid | 399.1257 | 2.6541 | C16H24O10 |
| 5 | Calycosin | 285.0753 | 7.3171 | C16H12O5 |
| 6 | Alpha-Linolenic acid | 279.2315 | 14.9590 | C18H30O2 |
| 7 | Aviprin | 305.1017 | 7.3171 | C16H16O6 |
| 8 | Benzoylhypacoitine | 574.2996 | 7.3865 | C31H43NO9 |
| 9 | Kanzonol O | 383.1482 | 10.9958 | C22H22O6 |
| 10 | Loganin | 413.1412 | 3.9643 | C17H26O10 |
| 11 | Sweroside | 381.1152 | 4.0555 | C16H22O9 |
| 12 | Tachiogroside B | 455.1156 | 3.5757 | C18H24O12 |
| 13 | Cuhuoside | 693.2385 | 7.0411 | C33H40O16 |
| 14 | 3-N-butyl-4,5-dihydrophthalide | 193.1217 | 10.4871 | C12H16O2 |
| 15 | Epimedokoreanoside II | 741.2352 | 10.2070 | C35H42O16 |
| 16 | Icariside II | 515.1902 | 10.2545 | C27H30O10 |
| 17 | Epimedin C | 845.2828 | 7.6841 | C39H50O19 |
| 18 | Epimedin B | 831.2675 | 7.5236 | C38H48O19 |
| 19 | Icariin | 677.2431 | 7.8434 | C33H40O15 |
| 20 | Ononin | 431.1330 | 6.8788 | C22H22O9 |
| 21 | Icariside E3 | 547.2141 | 5.0899 | C26H36O11 |
| 22 | Naringin | 603.1715 | 6.1112 | C27H32O14.Na |
| 23 | Lonijaposide A | 603.2018 | 6.1345 | C27H34NO13 |
| 24 | Hirsutrin | 487.0837 | 4.9511 | C21H20O12 |
| 25 | Isoscopoletin | 193.0487 | 4.9974 | C10H8O4 |
| 26 | Nodakenin | 409.1487 | 5.7864 | C20H24O9 |
| 27 | Violanthin | 579.1699 | 5.2764 | C27H30O14 |
| 28 | Axillarin 7-glucoside | 531.1103 | 5.9259 | C23H24O13 |
| 29 | Isofraxidin | 223.0595 | 5.3466 | C11H10O5 |
| 30 | Isorhamnetin 3-O-rutinoside | 647.1578 | 5.9025 | C28H32O16 |
| 31 | Circinasine B | 590.2940 | 6.7403 | C31H43NO10 |
| 32 | Brucine | 395.1953 | 4.1925 | C23H26N2O4 |
| 33 | Caulolactone A | 257.1511 | 13.8961 | C15H22O2 |
| 34 | Epimedoside E | 795.2718 | 6.4387 | C37H46O19 |
| 35 | (E)-Methyl-4-hydroxy-3-methoxycinnamate | 209.0804 | 9.4398 | C11H12O4 |
| 36 | Ikarisoside F | 633.2166 | 8.8602 | C31H36O14 |
| 37 | Formononetin | 269.0804 | 9.0917 | C16H12O4 |
| 38 | Anhydroicaritin 3-rhamnosyl-(1->2)-rhamnoside | 683.2296 | 9.9032 | C33H40O14 |
| 39 | Sagittatoside A | 699.2252 | 9.6480 | C33H40O15 |
| 40 | Oxypeucedanin | 287.0912 | 9.3002 | C16H14O5 |
| 41 | L-Phenylalanine | 166.0858 | 1.5838 | C9H11NO2 |
| 42 | trans-Cinnamic acid | 149.0589 | 11.7546 | C9H8O2 |
| 43 | Yesanchinoside F | 1173.6040 | 9.4627 | C56H94O24 |
| 44 | Campherenol | 223.2052 | 11.8464 | C15H26O |
| 45 | Pseudoginsenoside F8 | 1143.5917 | 9.5088 | C55H92O23 |
| 46 | Ginsenoside Rs3 | 849.4959 | 11.8464 | C44H74O14 |
| 47 | (+)-Sesamin | 337.1061 | 11.4094 | C20H18O6 |
| 48 | Glabridin | 325.1424 | 11.4094 | C20H20O4 |
| 49 | 6-Methylcoumarin | 161.1318 | 11.6631 | C10H8O2 |
| 50 | Ginsenoside Rd | 969.5388 | 9.4169 | C48H82O18 |
| 51 | Acanthol | 306.2786 | 12.0060 | C20H32O |
| 52 | Oleanolic acid 28-O-beta-D-glucopyranoside | 641.4014 | 11.9600 | C36H58O8 |
| 53 | Sapindoside B | 905.4860 | 10.5563 | C46H74O16 |
| 54 | 3'-Methoxyglabridin | 377.1353 | 10.6030 | C21H22O5 |
| 55 | Glyasperin C | 357.1687 | 10.4871 | C21H24O5 |
| 56 | Hemsgiganoside B | 979.4857 | 9.9032 | C48H76O19 |
| 57 | Licoagrochalcone D | 377.1350 | 10.2545 | C21H22O5 |
| 58 | Kumatakenin | 315.0861 | 10.2311 | C17H14O6 |
| 59 | Licoarylcoumarin | 391.1142 | 10.3473 | C21H20O6 |
| 60 | Palmitic acid | 274.2733 | 10.3008 | C16H32O2 |
| 61 | Carpusin | 303.0854 | 10.3240 | C16H14O6 |
| 62 | Gypenoside IX | 939.5270 | 10.0662 | C47H80O17 |
| 63 | gamma-Humulene | 205.1943 | 9.6712 | C15H24 |
| 64 | Ginsenoside Rg3 | 807.4856 | 11.1339 | C42H72O13 |
| 65 | Campesterol | 423.3604 | 18.7078 | C28H48O |
| 66 | epi-Lupeol | 427.3917 | 16.6453 | C30H50O |
| 67 | Shionone | 427.3555 | 17.2947 | C30H50O |
| 68 | Secosubamolide A | 391.2817 | 13.5486 | C22H40O4 |
| 69 | (-)-Falcarinol | 245.1892 | 12.7410 | C17H24O |
| 70 | Subamolide C | 337.2729 | 13.0403 | C21H36O3 |
| 71 | 2alpha,3alpha,19alpha,24-Tetrahydroxyurs-12-en-  28-oic acid 28-O-beta-D-glucopyranosyl ester | 689.3857 | 13.1100 | C36H58O11 |
| 72 | Patchouli alcohol | 205.1943 | 12.9940 | C15H24 |
| 73 | (-)-Globulol | 223.2048 | 13.8498 | C15H26O |
| 74 | Acanthopanaxoside C | 787.4213 | 15.1434 | C41H64O13 |
| 75 | Alpigenoside | 459.1465 | 3.9183 | C18H28O12 |
| 76 | Circinadine B | 454.2793 | 3.7127 | C24H39NO7 |
| 77 | Dryopteric acid | 349.0930 | 3.7813 | C17H16O8 |
| 78 | Phellodenol H | 369.1173 | 4.3071 | C17H20O9 |
| 79 | Circinasine F | 470.2737 | 3.3202 | C24H39NO8 |
| 80 | Secologanoside 7-methyl ester | 427.1206 | 3.4132 | C17H24O11 |
| 81 | Secologanic acid | 397.1105 | 3.4132 | C16H22O10 |
| 82 | Sempervirenoside B | 868.3230 | 3.3897 | C40H50O20 |
| 83 | Rocymosin B | 454.1704 | 3.1605 | C21H24O10 |
| 84 | Bullatine G | 358.2371 | 3.1375 | C22H31NO3 |
| 85 | Chalconaringenin 2'-O-glucoside 4'-O-gentobioside | 781.2134 | 3.2290 | C33H42O20 |
| 86 | Korolkoside | 827.2930 | 3.6670 | C36H52O20 |
| 87 | (2S)-7-[(2S,3R,4S,5S,6R)-4,5-dihydroxy-6-(hydroxymethyl)-3-[(2S,3R,4R,5R,6S)-3,4,5-trihydroxy-6-methyloxan-2-yl]oxyoxan-2-yl]oxy-2-(3,4-dihydroxyphenyl)-5-hydroxy-2,3-dihydrochromen-4-one | 619.1628 | 3.4365 | C27H32O15 |
| 88 | 5-Hydroxy-6-methoxy-7-O-beta-D-  glucosylcoumarin | 393.0785 | 3.5065 | C16H18O10 |
| 89 | Kelampayoside A | 501.1572 | 3.4832 | C20H30O13 |
| 90 | Psoralen | 187.0381 | 5.0669 | C11H6O3 |
| 91 | Neoline | 438.2842 | 4.9511 | C24H39NO6 |
| 92 | Quercetin 3-alpha-L-arabinofuranosyl-(1->2)-glucoside | 619.1260 | 4.8369 | C26H28O16 |
| 93 | Sophoraflavone B | 417.1169 | 4.8369 | C21H20O9 |
| 94 | Staunoside C | 619.2001 | 5.2999 | C28H36O14 |
| 95 | Phellodendroside | 519.1874 | 5.1833 | C26H30O11 |
| 96 | Phellodenol G | 431.1314 | 4.4680 | C20H24O9 |
| 97 | (S)-Reticuline | 330.1695 | 4.4450 | C19H23NO4 |
| 98 | Oligandrumin E | 349.1256 | 4.6071 | C16H22O7 |
| 99 | Hypoxanthine | 137.0453 | 0.9515 | C5H4N4O |
| 100 | Guanosine | 284.0994 | 0.9282 | C10H13N5O5 |
| 101 | o-Tyrosine | 182.0808 | 0.9052 | C9H11NO3 |
| 102 | Adenosine | 268.1042 | 0.9052 | C10H13N5O4 |
| 103 | Veranisatin E | 395.0953 | 1.3270 | C16H20O10 |
| 104 | Circinasine E | 424.2687 | 2.7462 | C23H37NO6 |
| 105 | N-Deethyldelphatine | 454.2793 | 2.9765 | C24H39NO7 |
| 106 | (S)-Isoboldine | 328.1540 | 2.9765 | C19H21NO4 |
| 107 | 2-methyl-3-[(2S,3R,4S,5S,6R)-3,4,5-trihydroxy-6-(hydroxymethyl)oxan-2-yl]oxypyran-4-one | 311.0737 | 1.6306 | C12H16O8 |
| 108 | Paniculamine | 394.2585 | 1.7941 | C22H35NO5 |
| 109 | Pseudoephedrine | 166.1222 | 2.3331 | C10H15NO |
| 110 | 7-Hydroxycoumarin | 163.0382 | 2.1703 | C9H6O3 |
| 111 | 5-Hydroxymethyl-2-furaldehyde | 127.0384 | 2.1703 | C6H6O3 |
| 112 | Cinnamyl alcohol | 152.1065 | 2.1238 | C9H10O |
| 113 | Linderane | 261.1117 | 8.0728 | C15H16O4 |
| 114 | Ludaconitine | 588.3157 | 7.9119 | C32H45NO9 |
| 115 | 8-Prenylcatechin | 381.1306 | 8.2831 | C20H22O6 |
| 116 | (+)-Alantolactone | 233.1528 | 7.4095 | C15H20O2 |
| 117 | 3-Oxopseudoanisatin | 319.1151 | 7.3640 | C15H20O6 |
| 118 | Dihydrobuddlenol B | 609.2292 | 7.3171 | C31H38O11 |
| 119 | Icariside C2 | 441.2449 | 7.6841 | C21H38O8 |
| 120 | Aconitine | 646.3202 | 7.6609 | C34H47NO11 |
| 121 | Hypaconitine | 616.3104 | 8.9064 | C33H45NO10 |
| 122 | Noranhydroicaritin 3-rhamnosyl-(1->2)-rhamnoside | 647.2331 | 8.8832 | C32H38O14 |
| 123 | Acankoreoside A | 979.4863 | 8.9769 | C48H76O19 |
| 124 | Licopyranocoumarin | 385.1273 | 8.8139 | C21H20O7 |
| 125 | Liquiritigenin | 257.0805 | 8.8139 | C15H12O4 |
| 126 | 14-O-Anisoylneoline | 572.3201 | 8.7906 | C32H45NO8 |
| 127 | (20R)-Ginsenoside RH1 | 661.4275 | 9.1610 | C36H62O9 |
| 128 | Parthenolide | 249.1477 | 9.1610 | C15H20O3 |
| 129 | Chasmaconitine | 614.3316 | 9.2537 | C34H47NO9 |
| 130 | 2alpha-Hydroxyursolic acid | 495.3459 | 9.2537 | C30H48O4 |
| 131 | Bidentatoside I | 977.4361 | 9.2304 | C47H70O20 |
| 132 | Ginsenoside Rh4 | 621.4352 | 8.9997 | C36H60O8 |
| 133 | Floralginsenoside M | 1101.5816 | 9.0227 | C53H90O22 |
| 134 | Licoriphenone | 373.1650 | 8.7671 | C21H24O6 |
| 135 | Alpinenone | 273.1456 | 8.5132 | C15H22O3 |
| 136 | Nipponoside D | 981.5013 | 8.4219 | C48H78O19 |
| 137 | Yesanchinoside E | 1131.5918 | 8.7209 | C54H92O23 |
| 138 | Gancaonin P | 371.1125 | 8.6509 | C20H18O7 |
| 139 | Xylostosidine | 416.1368 | 5.9489 | C18H25NO8S |
| 140 | Sylvestroside I | 771.2699 | 5.9489 | C33H48O19 |
| 141 | Tetrahydropalmatine | 356.1850 | 5.9719 | C21H25NO4 |
| 142 | Galangin | 271.0593 | 6.0179 | C15H10O5 |
| 143 | Perlolyrine | 265.0971 | 5.9025 | C16H12N2O2 |
| 144 | Dunnianin | 405.1895 | 6.1809 | C22H28O7 |
| 145 | Vitexin | 433.1123 | 6.1345 | C21H20O10 |
| 146 | Glycyrrhizol B | 368.1489 | 6.0879 | C21H18O5 |
| 147 | (2S,3S,4S,5R,6S)-6-[2-(3,4-dihydroxyphenyl)-5-hydroxy-4-oxochromen-7-yl]oxy-3,4,5-trihydroxyoxane-2-carboxylic acid | 463.0860 | 5.5071 | C21H18O12 |
| 148 | Astragalin | 449.1070 | 5.5301 | C21H20O11 |
| 149 | (2R)-2-(3,4-dihydroxyphenyl)-5,7-dihydroxy-2,3-dihydrochromen-4-one | 289.0703 | 5.5532 | C15H12O6 |
| 150 | Delsoline | 468.2948 | 5.3234 | C25H41NO7 |
| 151 | Hyperin | 465.1015 | 5.4152 | C21H20O12 |
| 152 | Phellochinin A | 519.1843 | 5.3924 | C26H30O11 |
| 153 | (+)-Marmesin | 247.0961 | 5.7864 | C14H14O4 |
| 154 | Dihydroprudomenin | 495.1474 | 5.5996 | C23H26O12 |
| 155 | Majoroside F2 | 823.4810 | 6.9020 | C42H72O14 |
| 156 | Liquiritigenin 4'-[3-acetylapiosyl-(1->2)-glucoside] | 593.1861 | 6.7860 | C28H32O14 |
| 157 | Shinpterocarpin | 340.1535 | 6.9718 | C20H18O4 |
| 158 | Berberine | 336.1222 | 7.1560 | C20H17NO4 |
| 159 | Palmatine | 352.1537 | 7.1105 | C21H22NO4 |
| 160 | Acanjaposide C | 965.4713 | 7.1560 | C47H74O19 |
| 161 | Isoliquiritigenin | 257.0805 | 6.9950 | C15H12O4 |
| 162 | Sylvestroside IV | 607.2009 | 6.9950 | C27H36O14 |
| 163 | Coumarin | 147.0433 | 6.3680 | C9H6O2 |
| 164 | (Z)-Aldosecologanin | 781.2522 | 6.4852 | C34H46O19 |
| 165 | Icariside E4 | 529.2042 | 6.3444 | C26H34O10 |
| 166 | Cantleyoside | 769.2531 | 6.3444 | C33H46O19 |
| 167 | Persiconin | 496.1812 | 6.3209 | C23H26O11 |
| 168 | Jatrorrhizine | 338.1381 | 6.5318 | C20H20NO4 |
| 169 | Notoginsenoside N | 985.5335 | 6.5780 | C48H82O19 |

Abbreviations here and below: RT, retention time.

**Table S3.** The compounds in WJT extract identified by UHPLC-QTOF-MS in negative mode.

| **NO.** | **Name** | **RT (min)** | **Mass (m/z)** | **Formula** |
| --- | --- | --- | --- | --- |
| 1 | Ginsenoside Rg1 | 6.9138 | 845.4916 | C42H72O14.HCOOH |
| 2 | Sweroside | 4.0392 | 403.1240 | C16H22O9 |
| 3 | Ginsenoside Rf | 8.5499 | 845.4917 | C42H72O14.HCOOH |
| 4 | 20(R)-Ginsenoside Rg3 | 11.1220 | 829.4962 | C42H72O13 |
| 5 | Neoeriocitrin | 5.5422 | 595.1668 | C27H32O15 |
| 6 | (E)-Aldosecologanin | 6.4828 | 757.2568 | C34H46O19 |
| 7 | Floralginsenoside M | 9.0415 | 1123.5924 | C53H90O22 |
| 8 | Chikusetsusaponin V | 8.9750 | 955.4922 | C48H76O19 |
| 9 | Liquiritin apioside | 6.5288 | 549.1613 | C26H30O13 |
| 10 | (2R,3R)-2,3-bis[(4-hydroxy-3-methoxyphenyl)methyl]butane-1,4-diol | 6.5288 | 361.1651 | C20H26O6 |
| 11 | Morroniside | 3.0050 | 451.1438 | C17H26O11 |
| 12 | Ginsenoside Rg2 | 8.9525 | 829.4964 | C42H72O13 |
| 13 | 3-Methyl-2-butenyl 6-O-alpha-L-arabinopyranosyl-beta-D-glucopyranoside | 3.4525 | 425.1653 | C16H28O10 |
| 14 | Cantleyoside | 6.3463 | 791.2643 | C33H46O19 |
| 15 | Ginsenoside Rg5 | 12.4698 | 811.4858 | C42H70O12 |
| 16 | Ginsenoside Rd | 9.3978 | 991.5500 | C48H82O18 |
| 17 | Ginsenoside R2 | 8.7512 | 815.4806 | C41H70O13 |
| 18 | 8-Epiloganic acid | 2.6468 | 375.1290 | C16H24O10 |
| 19 | Glycyroside | 6.7101 | 561.1618 | C27H30O13 |
| 20 | Sylvestroside I | 5.9444 | 793.2784 | C33H48O19 |
| 21 | Yesanchinoside E | 8.7067 | 1107.5965 | C54H92O23 |
| 22 | Naringin | 6.1014 | 579.1720 | C27H32O14.Na |
| 23 | Ginsenoside R1 | 6.6649 | 977.5344 | C47H80O18 |
| 24 | Ginsenoside Rs3 | 11.8432 | 871.5065 | C44H74O14 |
| 25 | Notoginsenoside R3 | 6.5516 | 1007.5454 | C48H82O19 |
| 26 | Epimedin A | 7.4080 | 883.2888 | C39H50O20 |
| 27 | Mangiferonic acid | 15.9143 | 453.3363 | C30H46O3 |
| 28 | Trehalose | 0.6345 | 341.1082 | C12H22O11 |
| 29 | Eleutheroside E | 5.0719 | 787.2680 | C34H46O18.HCOOH |
| 30 | Icariside E3 | 5.0719 | 523.2172 | C26H36O11 |
| 31 | Achyranthoside E | 9.4420 | 925.4458 | C46H70O19 |
| 32 | Ginsenoside Rh1 | 9.4200 | 683.4378 | C36H62O9 |
| 33 | Nonanal | 9.6429 | 187.1340 | C9H18O |
| 34 | Sagittatoside A | 9.6429 | 675.2300 | C33H40O15 |
| 35 | Glyasperin C | 10.4741 | 355.1541 | C21H24O5 |
| 36 | Licoflavone A | 10.4517 | 321.1132 | C20H18O4 |
| 37 | Catechin 5,7,4'-trimethyl ether | 9.2639 | 331.1190 | C18H20O6 |
| 38 | Bidentatoside I | 9.2191 | 953.4402 | C47H70O20 |
| 39 | Chikusetsusaponin IVa | 9.3978 | 793.4387 | C42H66O14 |
| 40 | Sapindoside B | 10.5409 | 881.4918 | C46H74O16 |
| 41 | Inokosterone | 9.4200 | 525.3062 | C27H44O7 |
| 42 | Glycyrrhizic acid | 9.4200 | 821.3978 | C42H62O16 |
| 43 | Subamolide E | 10.1145 | 311.1861 | C16H26O3 |
| 44 | beta-Ionone | 10.0460 | 237.1490 | C13H20O |
| 45 | Ikarisoside A | 10.2509 | 545.1663 | C26H28O10 |
| 46 | Icariside II | 10.2509 | 513.1760 | C27H30O10 |
| 47 | Epimedokoreanoside II | 10.2057 | 717.2407 | C35H42O16 |
| 48 | Licoleafol | 10.1827 | 371.1132 | C20H20O7 |
| 49 | Anisoxide | 10.4064 | 247.1339 | C14H18O |
| 50 | (s)-(+)-Abscisic acid | 9.7552 | 263.1284 | C15H20O4 |
| 51 | Osthenol | 9.9562 | 275.0925 | C14H14O3 |
| 52 | Wujiapioside B | 9.9562 | 941.5129 | C48H78O18 |
| 53 | Achyranthoside H | 9.9338 | 1057.5248 | C51H80O20 |
| 54 | (R)-(+)-7-Hydroxy-8-(2-hydroxy-3-methyl-3-butenyl)-2H-1-benzopyran-2-one | 10.3392 | 245.0815 | C14H14O4 |
| 55 | Semilicoisoflavone B | 10.3615 | 351.0865 | C20H16O6 |
| 56 | Anhydroicaritin 3-rhamnosyl-(1->2)-rhamnoside | 9.9117 | 659.2348 | C33H40O14 |
| 57 | Araloside A | 8.2125 | 971.4862 | C47H74O18 |
| 58 | Pseudoginsenoside RC1 | 8.1448 | 1033.5592 | C50H84O19 |
| 59 | Sagittatoside C | 8.1900 | 717.2405 | C35H42O16 |
| 60 | Loniceroside C | 7.8993 | 1073.5553 | C53H86O22 |
| 61 | Jiadifenin | 7.8550 | 337.0924 | C16H18O8 |
| 62 | Wanepimedoside A | 7.8993 | 677.2455 | C33H42O15 |
| 63 | Yesanchinoside D | 7.7658 | 887.5019 | C44H74O15 |
| 64 | Icariin | 7.8329 | 721.2357 | C33H40O15 |
| 65 | Hydroxywighteone | 8.1448 | 399.1084 | C20H18O6 |
| 66 | Naringenin | 8.0331 | 271.0607 | C15H12O5 |
| 67 | Ikarisoside F | 8.8630 | 631.2029 | C31H36O14 |
| 68 | Noranhydroicaritin 3-rhamnosyl-(1->2)-rhamnoside | 8.9077 | 645.2189 | C32H38O14 |
| 69 | Inermoside | 9.1080 | 1015.5132 | C49H78O19 |
| 70 | Acanjaposide B | 8.6170 | 969.4712 | C47H72O18 |
| 71 | Cinnamyl acetate | 8.5277 | 221.0819 | C11H12O2 |
| 72 | Aspidin | 8.7733 | 459.2012 | C25H32O8 |
| 73 | Phaseol | 8.7512 | 381.0981 | C20H16O5 |
| 74 | Liquiritigenin | 8.8178 | 255.0662 | C15H12O4 |
| 75 | Epimedokoreanoside I | 8.7957 | 967.3105 | C43H54O22 |
| 76 | Gancaonin P 3'methyl ether | 8.7289 | 383.1124 | C21H20O7 |
| 77 | Alpinenone | 8.6620 | 249.1489 | C15H22O3 |
| 78 | Gancanin M | 8.7067 | 397.1282 | C21H20O5 |
| 79 | 7-O-Methyllicoricidin | 13.9001 | 437.2331 | C27H34O5 |
| 80 | Gibberellin A95 | 12.8934 | 329.1390 | C19H22O5 |
| 81 | Gibberellin A120 | 12.8934 | 313.1440 | C19H22O4 |
| 82 | Gancaonin H | 12.6701 | 419.1501 | C25H24O6 |
| 83 | Isoangustone A | 13.0276 | 421.1657 | C25H26O6 |
| 84 | (Z)-Palmitoleic acid | 14.3728 | 299.2221 | C16H30O2 |
| 85 | Methyl octadecanoate | 14.3953 | 343.2845 | C19H38O2 |
| 86 | Ginsenoside Rs4 | 12.6701 | 853.4978 | C44H72O13 |
| 87 | 1,8-Heptadecadiene-4,6-diyne-3,10-diol | 14.7575 | 259.1701 | C17H24O2 |
| 88 | Kanzonol V | 12.6701 | 421.1649 | C24H24O4 |
| 89 | Gancaonin E | 11.2117 | 469.1858 | C25H28O6 |
| 90 | Ginsenoside Rg7 | 11.4574 | 799.4856 | C42H72O14 |
| 91 | Gancaonin A | 11.5029 | 351.1238 | C21H20O5 |
| 92 | Glabridin | 11.4126 | 323.1283 | C20H20O4 |
| 93 | Glabranin | 11.4126 | 369.1336 | C20H20O4 |
| 94 | 3-Hydroxyglabrol | 11.3679 | 407.1857 | C25H28O5 |
| 95 | 6'',6''-Dimethylpyraono[2'',3'':7,8]kaempferol 4'-methyl ether 3-rhamnoside | 11.3457 | 557.1674 | C27H28O10 |
| 96 | 8-Prenylnaringenin | 10.6974 | 385.1283 | C20H20O5 |
| 97 | 3-Hydroxymorindone | 10.7644 | 285.0766 | C15H10O6 |
| 98 | alpha-Santalol | 11.5482 | 265.1804 | C15H24O |
| 99 | Glabroisoflavanone B | 10.9879 | 397.1280 | C21H20O5 |
| 100 | Licoflavanone | 10.8759 | 339.1232 | C20H20O5 |
| 101 | Licoagrochalcone C | 10.9427 | 353.1385 | C21H22O5 |
| 102 | 4-Hydroxy-2,4'-dimethoxychalcone | 10.9204 | 283.0970 | C17H16O4 |
| 103 | Glisoflavanone | 12.2462 | 423.1804 | C25H28O6 |
| 104 | Licocoumarone | 12.1791 | 385.1294 | C20H20O5 |
| 105 | Glisoflavanone | 12.5593 | 423.1805 | C25H28O6 |
| 106 | Notoginsenoside R7 | 12.4474 | 667.4429 | C36H62O8 |
| 107 | Subamolide A | 11.7302 | 385.2587 | C20H36O4 |
| 108 | Integrifoliolin | 11.7529 | 261.1132 | C14H16O2 |
| 109 | Hexadecane-1,16-dioic acid | 11.7529 | 285.2069 | C16H30O4 |
| 110 | Glyinflanin F | 11.6614 | 469.1862 | C25H28O6 |
| 111 | Glyasperin B | 12.0006 | 369.1352 | C21H22O6 |
| 112 | Glabrol | 11.9333 | 391.1906 | C25H28O4 |
| 113 | Oleanolic acid 28-O-beta-D-glucopyranoside | 11.9333 | 663.4117 | C36H58O8 |
| 114 | Kanzonol W | 11.9110 | 335.0922 | C20H16O5 |
| 115 | Kanzonol Y | 11.7982 | 409.2008 | C25H30O5 |
| 116 | Loganin | 3.9499 | 435.1501 | C17H26O10 |
| 117 | Alpigenoside | 3.9050 | 481.1553 | C18H28O12 |
| 118 | Caffeic acid | 3.3175 | 179.0348 | C9H8O4 |
| 119 | Secologanic acid | 3.4075 | 373.1136 | C16H22O10 |
| 120 | 3-O-Caffeoylquinic acid | 3.1613 | 353.0874 | C16H18O9 |
| 121 | Aesculetin | 3.2507 | 177.0190 | C9H6O4 |
| 122 | Fraxin | 3.4975 | 369.0822 | C16H18O10 |
| 123 | Pseudomajucin | 4.6009 | 281.1390 | C15H22O5 |
| 124 | o-Coumaric acid | 4.5559 | 209.0457 | C9H8O3 |
| 125 | Hirsutrin | 4.9369 | 463.0874 | C21H20O12 |
| 126 | Sophoraflavone B | 4.8241 | 415.1026 | C21H20O9 |
| 127 | Quercetin 3-alpha-L-arabinofuranosyl-(1->2)-glucoside | 4.8241 | 595.1307 | C26H28O16 |
| 128 | Panasenoside | 4.1514 | 655.1530 | C27H30O16 |
| 129 | Phellodenol G | 4.4659 | 453.1395 | C20H24O9 |
| 130 | Succinic acid | 0.9700 | 117.0188 | C4H6O4 |
| 131 | Gallic acid | 1.1058 | 169.0142 | C7H6O5 |
| 132 | 2-methyl-3-[(2S,3R,4S,5S,6R)-3,4,5-trihydroxy-6-(hydroxymethyl)oxan-2-yl]oxypyran-4-one | 1.6496 | 333.0824 | C12H16O8 |
| 133 | L-Phenylalanine | 1.5817 | 164.0715 | C9H11NO2 |
| 134 | 5-(hydroxymethyl)furan-2-carbaldehyde | 1.4010 | 125.0240 | C6H6O3 |
| 135 | Pyruvic acid | 0.7908 | 133.0141 | C3H4O3 |
| 136 | 3,4-Dihydroxybenzaldehyde | 2.5799 | 137.0240 | C7H6O3 |
| 137 | 2-Isopropylmalic acid | 2.9605 | 175.0611 | C7H12O5 |
| 138 | Quinic acid | 2.9385 | 191.0561 | C7H12O6 |
| 139 | Tachioside | 2.0380 | 347.0976 | C13H18O8 |
| 140 | 2-Oxo-6-dehydroxyneoanisatin | 6.8225 | 355.1029 | C15H18O7 |
| 141 | Licorice glycoside C1 | 6.9587 | 725.2098 | C36H38O16 |
| 142 | Afzelechin | 6.8681 | 319.0818 | C15H14O5 |
| 143 | Isoononin | 6.8681 | 475.1237 | C22H22O9 |
| 144 | Licorice glycoside D1 | 6.8912 | 695.1989 | C35H36O15 |
| 145 | Licodione 2'-methyl ether | 6.8912 | 285.0766 | C16H14O5 |
| 146 | (2S)-Hydroxy-3,4-dehydroneomajucin | 6.4828 | 309.0981 | C15H18O7 |
| 147 | Epimedoside E | 6.4368 | 793.2564 | C37H46O19 |
| 148 | Afzelin | 6.4828 | 431.0975 | C21H20O10 |
| 149 | Liquiritin | 6.6878 | 417.1186 | C21H22O9 |
| 150 | Licorice glycoside E | 7.4527 | 692.1989 | C35H35NO14 |
| 151 | Ethyl caffeate | 7.6768 | 207.0660 | C11H12O4 |
| 152 | Catechin-3'-methyl ether | 7.6768 | 349.0921 | C16H16O6 |
| 153 | Icariside C3 | 7.6768 | 463.2532 | C21H38O8 |
| 154 | Epimedin C | 7.6768 | 867.2940 | C39H50O19 |
| 155 | Luteolin | 7.1828 | 285.0401 | C15H10O6 |
| 156 | Isoliquiritigenin | 7.0033 | 255.0661 | C15H12O4 |
| 157 | Xanthotoxol | 7.0708 | 201.0191 | C11H6O4 |
| 158 | Cuhuoside | 7.0485 | 737.2304 | C33H40O16 |
| 159 | Glepidotin A | 7.2725 | 383.1142 | C20H18O5 |
| 160 | Epimedoicarisoside A | 7.3408 | 523.1821 | C24H30O10 |
| 161 | Quercetin | 7.2053 | 301.0349 | C15H10O7 |
| 162 | Hyperin | 5.3867 | 463.0874 | C21H20O12 |
| 163 | Indole-3-carboxaldehyde | 5.6766 | 144.0452 | C9H7NO |
| 164 | Anhydrosafflor yellow B | 5.5201 | 1043.2690 | C48H52O26 |
| 165 | Trifolin | 5.5201 | 447.0926 | C21H20O11 |
| 166 | Kaempferol 3-(2'',3''-diacetylrhamnoside)-7-rhamnoside | 5.2751 | 707.1857 | C31H34O16 |
| 167 | Isoferulic acid | 5.0946 | 193.0504 | C10H10O4 |
| 168 | Chavicol beta-D-glucoside | 5.1627 | 341.1233 | C15H20O6 |
| 169 | Ikarisoside C | 6.3688 | 823.2680 | C38H48O20 |
| 170 | Anchoic acid | 6.3014 | 187.0974 | C9H16O4 |
| 171 | 20-Hydroxyecdysone | 5.7218 | 525.3069 | C27H44O7.HCOOH |
| 172 | Polypodine B | 5.7664 | 541.3013 | C27H44O8 |
| 173 | 6'''-(3-Hydroxy-3-methylglutaroyl)isoviolanthin | 5.7664 | 721.2000 | C33H38O18 |
| 174 | Nicotiflorin | 5.7443 | 593.1517 | C27H30O15 |
| 175 | Lisianthioside | 5.9669 | 761.2520 | C32H44O18 |
| 176 | Quercetin 3-O-L-rhamnoside | 5.9669 | 447.0925 | C21H20O11 |
| 177 | Isorhamnetin 3-O-rutinoside | 5.8774 | 623.1625 | C28H32O16 |

Abbreviations here and below: RT, retention time.

**Table S4.** Five major functions of 184 DEGs in three different categories of GO analysis.

| **Term** | **padj** | **Count** | **Gene Names** |
| --- | --- | --- | --- |
| **Biological process** | |  |  |
| chromosome segregation | 2.88E-15 | 25 | *BRCA1,BRIP1,BUB1,CENPC,CENPE,DDX3X,ECT2,ESCO2,GEN1,KIF18A,KIF4A,KNL1,NCAPG,PDS5A,PIBF1,SGO2,SLF1,SMC2,SMC3,SMC4,SMC5,SMC6,STAG2,TOP2A,TTK* |
| Nuclear chromosome  segregation | 1.44E-12 | 21 | *BRIP1,BUB1,CENPC,CENPE,ECT2,ESCO2,GEN1,KIF18A,KIF4A,KNL1,NCAPG,PDS5A,PIBF1,SGO2,SLF1,SMC2,SMC3,SMC4,SMC5,STAG2,TTK* |
| sister chromatid  segregation | 2.59E-12 | 18 | *BUB1,CENPC,CENPE,ESCO2,GEN1,KIF18A,KIF4A,NCAPG,PDS5A,PIBF1,SGO2,SLF1,SMC2,SMC3,SMC4,SMC5,STAG2,TTK* |
| organelle fission | 3.60E-10 | 23 | *BRCA2,BRIP1,BUB1,CAV2,CENPC,CENPE,GEN1,KIF11,KIF18A,KIF20B,KIF4A,NCAPG,PDS5A,PIBF1,SGO2,SLF1,SMC2,SMC3,SMC4,STAG1,STAG2,TTK,VPS35* |
| nuclear division | 3.60E-10 | 22 | *BRCA2,BRIP1,BUB1,CAV2,CENPC,CENPE,GEN1,KIF11,KIF18A,KIF20B,KIF4A,NCAPG,PDS5A,PIBF1,SGO2,SLF1,SMC2,SMC3,SMC4,STAG1,STAG2,TTK* |
| **Cell Component** |  |  |  |
| condensed chromosome | 1.42E-10 | 16 | *AHCTF1,BRCA1,BRCA2,BUB1,CENPC,CENPE,MIS18BP1,NCAPG,SMC2,SMC3,SMC4,SMC5,SMC6,STAG1,STAG2,TOP2A* |
| chromosomal region | 5.59E-08 | 16 | *AHCTF1,BRCA2,BUB1,CENPC,CENPE,CENPI,DHX36,ESCO2,FMR1,MIS18BP1,NCAPG,ORC4,SGO2,SMC6,SMCHD1,TTK* |
| mitotic spindle | 4.74E-07 | 10 | *CENPE,CEP295,ECT2,HAUS3,KIF11,KIF18A,KIF20B,SMC3,STAG1,STAG2* |
| chromosome, centromeric region | 7.58E-06 | 11 | *AHCTF1,BUB1,CENPC,CENPE,CENPI,ESCO2,FMR1,MIS18BP1,NCAPG,SGO2,TTK* |
| centrosome | 8.45E-06 | 18 | *BRCA2,CEP295,GEN1,HAUS3,HMMR,IFT74,KIF20B,LRRCC1,PCM1,PIBF1,PLK4,PPP4R3B,RANBP2,ROCK2,RPGRIP1L,SCLT1,SLF1,TRIM59* |
| **Molecular Function** | |  |  |
| ATPase activity | 4.69E-05 | 17 | *ABCA5,ATAD2,ATAD5,BRIP1,CENPE,DDX3X,DHX36,HLTF,KIF11,KIF18A,KIF20B,KIF4A,PMS1,RAD50,SMARCA5,SMCHD1,TOP2A* |
| tubulin binding | 0.0004745 | 13 | *BRCA2,CENPE,CEP295,FCHO2,FMR1,IFT74,KIF11,KIF15,KIF18A,KIF20B,KIF4A,SMC3,VPS41* |
| microtubule binding | 0.0038187 | 10 | *CENPE,CEP295,FCHO2,FMR1,KIF11,KIF15,KIF18A,KIF20B,KIF4A,VPS41* |
| microtubule motor activity | 0.0076068 | 6 | *CENPE,KIF11,KIF15,KIF18A,KIF20B,KIF4A* |
| motor activity | 0.0118983 | 7 | *CENPE,KIF11,KIF15,KIF18A,KIF20B,KIF4A,MYO9A* |

**Table S5.** Ten major KEGG pathways among the 184 DEGs.

| **Description** | **padj** | **Count** | **Gene Names** |
| --- | --- | --- | --- |
| Fanconi anemia pathway | 0.014806 | 5 | *ATR,BRCA1,BRCA2,BRIP1,FANCB* |
| Cell cycle | 0.018226 | 7 | *ATR,BUB1,ORC4,SMC3,STAG1,STAG2,TTK* |
| Homologous recombination | 0.037527 | 4 | *BRCA1,BRCA2,BRIP1,RAD50* |
| RNA transport | 0.168084 | 6 | *EIF3A,FMR1,FXR1,THOC1,TRNT1,UPF3B* |
| Platinum drug resistance | 0.168084 | 4 | *BRCA1,PMAIP1,TOP2A,XIAP* |
| Non-homologous end-joining | 0.168084 | 2 | *RAD50,XRCC4* |
| Mineral absorption | 0.168084 | 3 | *FXYD2,HMOX1,TRPM7* |
| Ubiquitin mediated proteolysis | 0.219722 | 5 | *BRCA1,CUL4B,UBA6,UBE3A,XIAP* |
| Apoptosis - multiple species | 0.637965 | 2 | *PMAIP1,XIAP* |
| Fructose and mannose metabolism | 0.637965 | 2 | *AKR1B10,FPGT* |

Abbreviations here and below: padj, *p*-value adjusted for multiple testing.

**Table S6.** Ten major KEGG terms based on DEGs in the Turquoise module.

| **Term** | **padj** | **Count** | **Gene Names** |
| --- | --- | --- | --- |
| Propanoate metabolism | 0.351154 | 3 | *DBT,DLD,SUCLA2* |
| Colorectal cancer | 0.363354 | 4 | *CASP3,PMAIP1,RPS6KB1,TGFBR1* |
| Human immunodeficiency virus 1 infection | 0.363354 | 6 | *AP1S3,CASP3,CUL4B,GNAI3,RPS6KB1,*  *TRIM5* |
| Insulin signaling pathway | 0.532016 | 4 | *HK3,PPP1CB,PRKAA1,RPS6KB1* |
| Apelin signaling pathway | 0.532016 | 4 | *GNAI3,PRKAA1,RPS6KB1,TGFBR1* |
| Citrate cycle (TCA cycle) | 0.532016 | 2 | *DLD,SUCLA2* |
| Oxytocin signaling pathway | 0.532016 | 4 | *GNAI3,PLA2G4A,PPP1CB,PRKAA1* |
| Apoptosis - multiple species | 0.532016 | 2 | *CASP3,PMAIP1* |
| Fructose and mannose metabolism | 0.532016 | 2 | *FPGT,HK3* |
| mRNA surveillance pathway | 0.532016 | 3 | *CPSF2,PPP1CB,UPF3B* |

Abbreviations here and below: padj, *p*-value adjusted for multiple testing.

**Table S7.** Ten major KEGG terms of DEGs in the Blue module.

| **Term** | **padj** | **Count** | **Gene Names** |
| --- | --- | --- | --- |
| RNA transport | 0.005212 | 7 | *EIF3A,EIF3J,NUP153,PNN,TGS1,*  *THOC1,TRNT1* |
| Cell cycle | 0.006535 | 6 | *ATR,BUB1,ORC2,ORC4,SMC3,TTK* |
| Homologous recombination | 0.074604 | 3 | *BRCA1,BRIP1,RAD50* |
| Fanconi anemia pathway | 0.078452 | 3 | *ATR,BRCA1,BRIP1* |
| Oocyte meiosis | 0.09399 | 4 | *BUB1,RPS6KA3,SGO1,SMC3* |
| Spliceosome | 0.716467 | 3 | *DDX46,PRPF40A,THOC1* |
| Non-homologous end-joining | 0.716467 | 1 | *RAD50* |
| Platinum drug resistance | 0.716467 | 2 | *BRCA1,TOP2A* |
| ECM-receptor interaction | 0.716467 | 2 | *HMMR,ITGA6* |
| Progesterone-mediated oocyte maturation | 0.716467 | 2 | *BUB1,RPS6KA3* |

Abbreviations here and below: padj, *p*-value adjusted for multiple testing.
